# Reconstitution of DNA Strand Exchange Mediated by Rhp51 Recombinase and Two Mediators

**DOI:** 10.1371/journal.pbio.0060088

**Published:** 2008-04-15

**Authors:** Yumiko Kurokawa, Yasuto Murayama, Nami Haruta-Takahashi, Itaru Urabe, Hiroshi Iwasaki

**Affiliations:** 1 Division of Molecular and Cellular Biology, International Graduate School of Arts and Sciences, Yokohama City University, Yokohama, Kanagawa, Japan; 2 Department of Biotechnology, Graduate School of Engineering, Osaka University, Suita, Osaka, Japan; Brandeis University, United States of America

## Abstract

In the fission yeast Schizosaccharomyces pombe, genetic evidence suggests that two mediators, Rad22 (the *S. pombe* Rad52 homolog) and the Swi5-Sfr1 complex, participate in a common pathway of Rhp51 (the S. pombe Rad51 homolog)–mediated homologous recombination (HR) and HR repair. Here, we have demonstrated an in vitro reconstitution of the central step of DNA strand exchange during HR. Our system consists entirely of homogeneously purified proteins, including Rhp51, the two mediators, and replication protein A (RPA), which reflects genetic requirements in vivo. Using this system, we present the first robust biochemical evidence that concerted action of the two mediators directs the loading of Rhp51 onto single-stranded DNA (ssDNA) precoated with RPA. Dissection of the reaction reveals that Rad22 overcomes the inhibitory effect of RPA on Rhp51-Swi5-Sfr1–mediated strand exchange. In addition, Rad22 negates the requirement for a strict order of protein addition to the in vitro system. However, despite the presence of Rad22, Swi5-Sfr1 is still essential for strand exchange. Importantly, Rhp51, but neither Rad22 nor the Swi5-Sfr1 mediator, is the factor that displaces RPA from ssDNA. Swi5-Sfr1 stabilizes Rhp51-ssDNA filaments in an ATP-dependent manner, and this stabilization is correlated with activation of Rhp51 for the strand exchange reaction. Rad22 alone cannot activate the Rhp51 presynaptic filament. AMP-PNP, a nonhydrolyzable ATP analog, induces a similar stabilization of Rhp51, but this stabilization is independent of Swi5-Sfr1. However, hydrolysis of ATP is required for processive strand transfer, which results in the formation of a long heteroduplex. Our in vitro reconstitution system has revealed that the two mediators have indispensable, but distinct, roles for mediating Rhp51 loading onto RPA-precoated ssDNA

## Author Summary

Homologous recombination promotes genetic diversity in the next generation and serves as a driving force for evolution. It also provides efficient machinery for repairing DNA damage such as double-strand breaks. Homologous recombination involves DNA exchange between homologous chromosomes, which is mediated by evolutionarily conserved proteins called recombinases. It is thought that a recombinase binds to single-stranded DNA (ssDNA) to form a nucleoprotein filament called the presynaptic filament, and that this higher order structure engages in a search for homologous DNA sequences. Once a homologous duplex is found, the presynaptic filament initiates strand exchange. However, when ssDNA regions are created, they are immediately covered by replication protein A (RPA), thereby inhibiting recombinase filament formation even under conditions in which homologous recombination is appropriate. Previous studies suggested that mediator proteins help load recombinases onto ssDNA, and further studies showed that at least two mediators function together in a single recombination pathway. How these mediators coordinate recombinase loading has been unclear. We have addressed this question by reconstituting an in vitro strand exchange reaction with purified proteins including a fission yeast recombinase, Rhp51, two mediators, Rad22 and the Swi5-Sfr1 complex, and RPA. Our results indicate that Rad22 orchestrates the loading of Rhp51 onto RPA-coated ssDNA by acting as a scaffold for nucleating the recombinase filament, whereas the other mediator, Swi5-Sfr1, stabilizes and activates the filament.

## Abstract 3

A test tube reconstitution of the strand exchange reaction provides further evidence that the two mediators cooperatively promote the nucleation of active recombinase-ssDNA filaments, creating a higher order structure for recombination.

## Introduction

Homologous recombination (HR) generates genetic diversity by rearranging DNA sequences using homologous DNA information. It is also an important mechanism for repairing DNA double-stranded breaks (DSBs) and restarting stalled DNA replication forks. Accordingly, HR is essential to the preservation of genome integrity; defects in HR result in hypersensitivity to genotoxic agents and chromosomal aberrations. One conspicuous example of the role of HR in genome integrity is cancer prevention via the tumor suppressors BRCA1 and BRCA2, both of which interact with the Rad51 recombinase [1].

Genetic analyses of DSB repair and mitotic and meiotic HR in the budding yeast Saccharomyces cerevisiae have revealed a major pathway for HR that is under the control of proteins in the Rad52 epistasis group [2–4]. The Rad51 recombinase belongs to the Rad52 group and plays a key role in HR: Rad51 forms nucleoprotein filaments with single-stranded DNA (ssDNA), referred to as presynaptic filaments, and promotes strand exchange with donor DNA in an ATP-dependent manner. A series of analyses suggested that the assembly pathway for Rad51 on ssDNA in vivo is spatiotemporally regulated by replication protein A (RPA) and other Rad52 epistasis group proteins, such as Rad52 and Rad55–57 [5,6]. RPA immediately binds to ssDNA regions once they are formed (e.g., by the resection of DSB ends or by stalled replication forks). Rad51 alone cannot bind to RPA-coated ssDNA, as RPA has higher affinity for ssDNA than does the Rad51 recombinase. Rad52 assists in loading Rad51 onto RPA-coated ssDNA and in assembling the Rad51 nucleoprotein filament. The Rad51 paralogs Rad55 and Rad57, which form a heterodimer, assist Rad51-mediated filament assembly and/or stabilize the filament, leading to efficient strand exchange [7,8]. Proteins that facilitate Rad51 loading or filament stabilization are referred to as mediators [3,9]. The basic characteristics of the early steps of HR are widely conserved among eukaryotes; however, multicellular eukaryotes, including humans, have five Rad51 paralogs, XRCC2, XRCC3, RAD51B, RAD51C, and RAD51D. Several Rad51 paralog complexes have been observed, and these are also assumed to function as mediators [10,11]. In addition, BRCA2, a tumor suppressor, has been suggested to act as a recombination mediator [12–15].

The fission yeast Schizosaccharomyces pombe, which is evolutionarily distant from S. cerevisiae, uses an HR pathway very similar to that of budding yeast. However, a notable exception in S. pombe is the Swi5-Sfr1 complex, which functions as an additional mediator in the HR pathway involving Rad22 and Rhp51 (fission yeast Rad52 and Rad51 homologs, respectively) (reviewed in [16]). The Swi5-Sfr1 complex operates in the Rhp51-dependent HR pathway in parallel with another mediator, the Rhp55-Rhp57 complex (fission yeast Rad55 and Rad57 homologs, respectively) in vivo [17,18].

Swi5 is a small protein that is evolutionarily conserved from S. cerevisiae to man, but it has no known protein motifs [17,19,20]. Sfr1 (Swi5-dependent recombination repair protein 1) was identified as a Swi5 interactor that is involved in HR repair [17]. It shares homology with the C-terminal half of Swi2, which overlaps the interaction region for Swi5 and Rhp51 [17], and this region is modestly conserved from S. cerevisiae to man [20]. Sfr1 also lacks known functional motifs.

S. cerevisiae Sae3 and Mei5 are Swi5 and Sfr1 homologs, respectively. However, unlike S. pombe Swi5 and Sfr1, Sae3 and Mei5 are expressed only during meiosis. *sae3* and *mei5* mutants both show meiotic phenotypes very similar to those of the *S. cerevisiae dmc1* mutant. These mutant phenotypes and the cellular localization of the two proteins suggest that they are specific for meiotic recombination associated with the meiosis-specific recombinase Dmc1 [20,21]. On the other hand, *swi5* mutants exhibit more severe meiotic defects than do *dmc1* mutants. Thus, S. pombe Swi5 clearly has an additional function beyond that involved in Dmc1-dependent activities [19].

We recently purified the Swi5-Sfr1 complex and found that it has ssDNA and double-stranded DNA (dsDNA) binding activities but that it lacks nuclease, helicase, and ATPase activities [22]. Consistent with genetic studies, the purified Swi5-Sfr1 complex stimulates both Rhp51- and Dmc1-mediated strand exchange in vitro [22] (reviewed in [16]). The stimulation of Rhp51-mediated strand exchange is closely related to its ssDNA-dependent ATPase activity, which is enhanced by the Swi5-Sfr1 complex. The Swi5-Sfr1 complex does not enhance the binding of Rhp51 to ssDNA per se. On the other hand, the mediator enhances the binding of Dmc1 to ssDNA. The molecular bases of the different effects of Swi5-Sfr1 on the two recombinases are still unknown.

An important issue emerged from our previous study. The Swi5-Sfr1 complex cannot efficiently overcome the inhibitory effect of RPA when RPA is bound to ssDNA prior to Rhp51 binding. This observation is consistent with the observation that the Swi5-Sfr1 complex does not appreciably affect the ssDNA binding capacity of Rhp51. However, the canonical definition of a recombination mediator is that it is an ancillary factor that overcomes the inhibitory effect of RPA on a recombinase.

S. cerevisiae Rad52 protein has been shown to interact directly with both RPA and Rad51 and to promote Rad51 filament formation by mediating the displacement of prebound RPA from ssDNA, leading to effective strand exchange mediated by Rad51 [23–25]. Therefore, it is possible that Rad22 acts exclusively to overcome the inhibitory effect of RPA and that the Swi5-Sfr1 complex acts exclusively to activate Rhp51 filaments. Thus, the concerted actions of these two mediators, Rad22 and the Swi5-Sfr1 complex, would direct the loading of Rhp51 onto ssDNA, leading to efficient strand exchange.

The work described here addresses this hypothesis with an in vitro system that we have established, which reconstitutes the early central step of homologous recombination. We found that Rad22 overcomes the inhibitory effect of RPA on strand exchange mediated by Rhp51-Swi5-Sfr1, as predicted. However, Swi5-Sfr1 is still essential for strand exchange, and both Rad22 and Swi5-Sfr1 are required for full reaction efficiency. In-depth analysis indicates that the two mediators work concertedly, but not exclusively, by different effects on Rhp51 to form the active filament required for effective DNA strand exchange. In addition, we have shown that the Swi5-Sfr1 mediator stabilizes and activates Rhp51-ssDNA filaments in an ATP-dependent manner, whereas Rad22 is not involved in Rhp51 activation.

## Results

### Rad22 Overcomes the Inhibitory Effect of RPA, but Not of Bacterial ssDNA Binding Protein, on Strand Exchange Mediated by Rhp51-Swi5-Sfr1

We first determined whether purified Rad22 can overcome the inhibitory effect of RPA on strand exchange mediated by Rhp51-Swi5-Sfr1. Figure 1A shows a schematic diagram of the Rhp51-mediated three-strand exchange assay used in this study, in which pairing of a (+) strand DNA (circular ssDNA [css]) with a homologous linear duplex DNA (linear dsDNA [lds]) derived from øX174 phage generates a joint molecule (JM) that is converted to nicked circular DNA (NC) and linear ssDNA products by strand exchange. S. pombe Rad22 was bacterially expressed and purified as described in Materials and Methods. As previously reported [22], when css was first incubated with Rhp51 (and the Swi5-Sfr1 mediator) and then with RPA, large amounts of JMs and NCs were produced (Figure 1B). RPA was essential for strand exchange (compare lanes 1 and 2 in Figure 1B). In contrast, when css was first incubated with RPA and then with Rhp51 and Swi5-Sfr1, JM and NC formation was dramatically reduced (Figure 1B, lane 4), as previously reported [22]. This inhibitory effect of RPA was blocked by the addition of purified Rad22 (Figure 1B, lane5).

**Figure 1:**
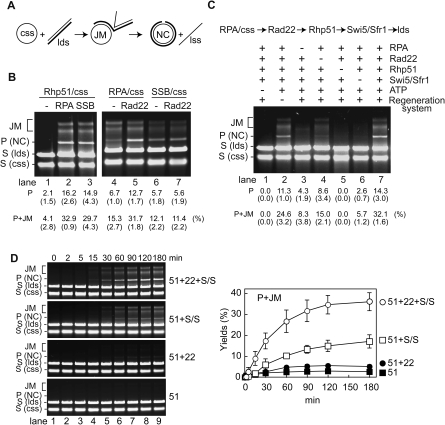
In Vitro Reconstitution of DNA Strand Exchange (A) Diagram of the DNA strand exchange assay. lss, linear single-stranded DNA. (B) Rad22 can overcome the inhibitory effect of RPA on the Rhp51-Swi5-Sfr1–mediated DNA strand exchange reaction. In lanes 1 to 3, Rhp51 was first incubated with css, and then with Swi5-Sfr1, RPA, and lds. In the lane 1 reaction, SSB was omitted. In lanes 2 and 3, RPA and SSB were added, respectively. In lanes 4 and 5, RPA was first incubated with css and then with Rhp51 and Swi5-Sfr1. Rad22 (lane 5) or mock buffer (lane 4) were added 5 min after incubation with RPA. Lanes 6 and 7 are the same as lanes 4 and 5, respectively, except that SSB was used instead of RPA. (C) Requirements for strand exchange. All protein cofactors are required for the reaction. The protein addition order is indicated above the agarose gel image. When a component was not included, it was replaced by stock buffer. The values indicated below each lane in (B) and (C) are average percentages of NC products (P) and conversions (P + JM) obtained in three independent experiments. Standard deviation (s.d.) is indicated in parentheses. (D) Time course of the strand exchange reaction with various protein components as indicated to the right of each gel image. The reaction procedures including the addition order were the same as in (C) except that the reaction volume was 100 μl. Aliquots (10 μl) were taken at various time points, and the reactions were terminated by adding a stop solution, with a final incubation for 30 min at 37 °C. The graph shows quantifications of P + JM. The values and error bars are the average percentage and s.d. of results from three independent experiments. 22, Rad22; 51, Rhp51; S/S, Swi5-Sfr1.

A roughly equivalent amount of bacterial ssDNA binding protein (SSB; 2 μM) could be substituted for RPA (1 μM) when the strand exchange reaction was initiated by Rhp51/css complex formation (Figure 1B, lane 3). This result is consistent with RPA acting to prevent reversal of the already formed DNA joints by sequestering the free ssDNA, a reaction in which RPA can be replaced by SSB. However, when css was precoated with SSB (2 μM) before adding Rhp51 and Swi5-Sfr1, JM and NC product formation was severely reduced (Figure 1B, lane 6). More importantly, Rad22 could not overcome the inhibitory effect of SSB (Figure 1B, lane 7), indicating that functional interactions between RPA with Rad22 are important for this step.

RPA-coated ssDNA is assumed to be a natural substrate for the in vivo strand exchange reaction. Therefore, the requirements for reactions initiated with RPA-coated ssDNA were examined (Figure 1C). These reactions were strictly dependent on Rhp51, Swi5-Sfr1, RPA, and ATP. Rad22 was not essential, but in its absence, the levels of JM and NC products were severely reduced. Time-course experiments clearly demonstrated that Rad22 alone stimulated very little Rhp51-dependent strand exchange when RPA-coated ssDNA was used for a substrate (Figure 1D). Swi5-Sfr1 alone stimulated the reaction, and reactions in which Rad22 and Swi5-Sfr1 were coincubated with Rhp51 proceeded with robust efficiency. These results clearly indicate that full reaction efficiency requires the functions of the two mediators, Rad22 and Swi5-Sfr1.

### Rad22 Coordinates Strand Exchange

Next, we examined whether the timing of Rad22 addition affects the strand exchange reaction (Figure 2A), since the addition order is critical for the Rhp51-Swi5-Sfr1–mediated reaction [22]. Note that protocol 2 in Figure 2A employs the same addition order as that of the standard reaction (e.g., the reaction shown in Figure 1B). Surprisingly, the time at which Rad22 was added was not crucial (Figure 2A): all protocols were highly efficient, with the exception of reactions lacking Rad22. These results indicate that Rad22 can overcome the inhibitory action of RPA irrespective of when it is added.

**Figure 2:**
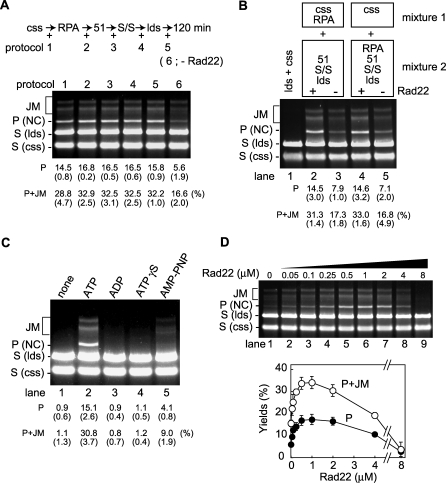
Rad22 Coordinates Strand Exchange (A) Effect of the order of Rad22 addition. Various protocols, indicated by numbers, were tested (upper panel). Rad22 was added at the time point indicated by the plus sign (+). The incubation time of each step (arrows) was 5 min. Rad22 was added 2.5 min after the beginning of each indicated step. Samples from each of the reactions (lower panel) were separated by agarose gel electrophoresis. Protocols 1 to 5 gave similarly high levels of strand exchange. (B) Rad22 abrogates the strict requirement for a specific order of protein addition to the strand exchange reaction. The indicated mixtures (1 and 2) were prepared separately, and the reactions were initiated by combining both mixtures and incubating at 37 °C for 120 min. (C) ATP hydrolysis is required for Rhp51-mediated strand exchange. The reactions contained Rad22, Swi5-Sfr1, Rhp51 and RPA, and the indicated nucleotides. The reaction conditions were the same as in Figure 1C. The values indicated below each lane in (A) to (C) are average percentages of P and P + JM obtained in three independent experiments. The s.d. is indicated in parentheses. (D) Substoichiometric amounts of Rad22 stimulate Rhp51-mediated DNA strand exchange. An agarose gel containing reactions with increasing concentrations of Rad22 is shown in the upper panel. The graph below shows the yields of the NC product (filled circles) and the total yields of JM intermediates plus the NC product (open circles). The values and error bars are average percentages and s.d. obtained from three independent experiments.

These data suggest that Rad22 may coordinate strand exchange in a single mixture that includes all protein components. To address this possibility, we set up the following reactions. We prepared two mixtures, one containing RPA-coated css and the other containing all other reaction constituents. The reactions were started by combining the two mixtures and incubating at 37 °C for 120 min. As shown in Figure 2B, the results clearly indicate that this protocol allows fully efficient reactions initiated from RPA-coated ssDNA and that Rad22 is essential, since reaction efficiency was substantially decreased if it was omitted (compare lanes 2 and 3 in Figure 2B). Furthermore, we found that a mixture containing all protein components (RPA, Rad22, Rhp51, and Swi5-Sfr1) efficiently promoted strand exchange when combined with css (Figure 2B, lane 4). Rad22 is essential for this reaction as well (compare lanes 4 and 5). These results indicate that Rad22 coordinates the functions of all proteins and orchestrates DNA strand exchange in vitro.

The Rad22-dependent reactions required ATP hydrolysis. ADP or ATPγS did not promote strand exchange (Figure 2C), as previously observed for the Swi5-Sfr1–dependent reaction in the absence of Rad22 [22]. Interestingly, AMP-PNP supported a small amount of JM formation, but not NC production. The most effective concentration of Rad22 was approximately one tenth the concentration of Rhp51. Higher concentrations of Rad22 inhibited the reaction (Figure 2D).

### The Two Mediators Function Synergistically to Promote Rhp51 Loading onto ssDNA

The inhibitory effect of RPA may result from its higher affinity for ssDNA compared to that of Rhp51. If this is correct, either or both of the two mediators may function to displace RPA from ssDNA to facilitate the loading of Rhp51, thereby leading to efficient presynaptic filament formation for the strand exchange reaction. In addition, once the correct filament is formed, it should be stabilized to protect against further RPA binding to ssDNA, since the thermodynamic equilibrium favors RPA binding to ssDNA. Either or both of the two mediators may be involved in this stabilization, as well.

To test these hypotheses, we set up pull-down assays in which the conditions were the same as for the strand exchange reaction. We first performed a titration experiment of RPA to css-bound beads (css beads) (10 μM nucleotides). The results of this experiment indicated that approximately 30% of input RPA (1 μM) was excess to RPA bound (∼0.7μM) to css beads (Figure 3A). Next, we analyzed the nucleotide dependency of Rhp51 binding to ssDNA (Figure 3B and 3C). In the absence of adenine nucleotides, Rhp51 did not bind to ssDNA, indicating that the ssDNA binding activity of Rhp51 requires an adenine nucleotide. Titration experiments indicated that both ATP and AMP-PNP were highly efficient cofactors, whereas ADP and ATPγS were slightly less effective (Figure 3B). Note that the amount of Rhp51 (5 μM) used in the standard strand exchange reaction is excessive (about 2.5- to 3-fold) to its binding to css beads in the presence of any adenine nucleotide.

**Figure 3:**
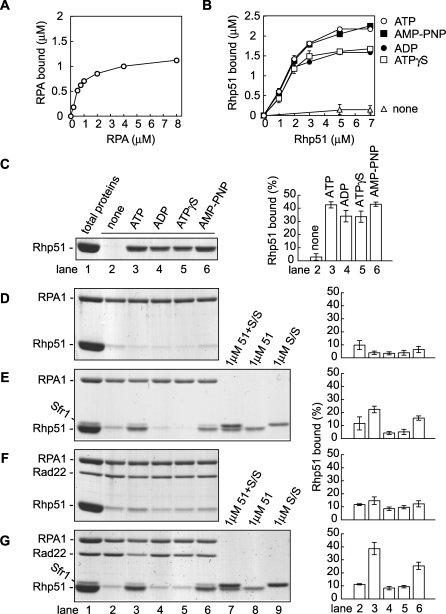
Pull-Down Assays Reveal That the Two Mediators Together Facilitate Rhp51 Presynaptic Filament Formation (A) A titration of RPA to css immobilized on magnetic beads. The indicated amounts of RPA were incubated with css beads (10 μM ssDNA) at 37 °C for 5 min. The protein–DNA complexes were pulled down, and RPA bound to ssDNA was analyzed by SDS-PAGE. (B) Titration of Rhp51 to css beads. The indicated amounts of Rhp51 were incubated with css beads (10 μM ssDNA) at 37 °C for 5 min in the absence or presence of various adenine nucleotides (2 mM). Rhp51-ssDNA complexes were pulled down, and Rhp51 bound to ssDNA was analyzed by SDS-PAGE. The values and error bars in the graphs in (A) and (B) are average percentages and s.d., respectively, obtained from three independent experiments. (C) Adenine nucleotide-dependent ssDNA binding of Rhp51. Rhp51 (5 μM) was pulled down with css beads (10 μM ssDNA) in the absence or presence of various adenine nucleotides (2 mM). The SDS-PAGE image shows bands corresponding to the Rhp51-ssDNA complex in the pull-down fractions. (D–G) RPA has a higher affinity than Rhp51 for ssDNA, and the two mediators synergistically promote Rhp51 presynaptic filament formation in an ATP-dependent manner. Mixtures, indicated below, were prepared and incubated with immobilized ssDNA. Pull-down complexes were analyzed by SDS-PAGE. (D) Rhp51 and RPA. (E) Rhp51, RPA, and Swi5-Sfr1. (F) Rhp51, RPA, and Rad22. (G) Rhp51, RPA, Swi5-Sfr1, and Rad22. The concentrations of proteins used in all assays were 5 μM Rhp51, 1 μM RPA, 0.5 μM Swi5-Sfr1, and 0.5 μM Rad22. A quantitation of bound Rhp51, calculated as a percentage of the amount of Rhp51 in each lane 1 (100%), is shown at right. The values and error bars in the graphs are average percentages and s.d., respectively, obtained from three independent experiments. (C–G) lane 1: input proteins, lane 2: no nucleotide, lane 3: ATP, lane 4: ADP, lane 5: ATPγS, and lane 6: AMP-PNP. Lanes 7, 8, and 9 in (E) and (G) contain Rhp51 (1μM) plus Swi5-Sfr1 (1μM), Rhp51 (1μM) alone, and Swi5-Sfr1 (1μM) alone, respectively, for standards of relative migrations of these proteins.

We then performed competition experiments to compare the ssDNA binding activity of Rhp51 to that of RPA. Mixtures containing RPA (1 μM) and Rhp51 (5 μM) were incubated with css beads. Protein–DNA complexes were pulled down, and the amount of Rhp51 bound to ssDNA was analyzed by SDS-PAGE (Figure 3D–3G). In the presence of adenine nucleotides, but in the absence of mediators, Rhp51 was not pulled down with css, indicating that RPA indeed has a higher affinity for ssDNA than does Rhp51 (Figure 3D). A similar result has been reported for Rad51 and RPA from budding yeast [26].

We examined which of the two mediators assists Rhp51 loading onto ssDNA. The Swi5-Sfr1 complex alone promoted Rhp51 loading onto ssDNA in the presence of ATP and AMP-PNP (Figure 3E). About 22% and 16% of the input Rhp51 were pulled down with ssDNA in the presence of ATP and AMP-PNP, respectively. Reactions containing ADP or ATPγS or lacking an adenine nucleotide only weakly supported Rhp51 loading (about 5% of the input Rhp51 was pulled down).

Incubation with Rad22 alone allowed a small amount of Rhp51 to be pulled down (about 10%) in the absence of a nucleotide cofactor or in the presence of ATP, ADP, ATPγS, or AMP-PNP (Figure 3F). The amounts of Rhp51 that were pulled down were not significantly affected by the absence or presence of the nucleotide cofactor, or by the type of nucleotide in this case. Budding yeast Rad52 associates with RPA-ssDNA to accelerate the Rad51-mediated displacement of RPA [27]. However, since the binding of Rhp51 to ssDNA required an adenine nucleotide (Figure 3B and 3C), the detection of Rhp51 in the pulled-down ssDNA protein complexes may reflect Rhp51 bound to Rad22 that is associated with preformed RPA-ssDNA complexes, rather than the direct binding of Rhp51 to ssDNA. The very low level of Rhp51 pulled down in the presence of ADP and ATPγS in the absence of Rad22 (Figure 3D and 3E, lanes 4 and 5) suggests that this basal level of Rhp51 is dependent on Rad22. We also observed Rhp51–Rad22 and Rad22–RPA interactions in the absence of an adenine nucleotide with an immunoprecipitation assay (unpublished data); similarly, tight Rad51–Rad52 and Rad52–RPA interactions have been reported for budding yeast and human cells [23–30]. These observations also support the notion that the small amount of Rhp51 pulled down when Rad22 alone is included is due to the basal level of Rhp51 that binds to Rad22, which in turn is associated with RPA bound to css beads.

When Rad22 and Swi5-Sfr1 were present, Rhp51 was loaded onto ssDNA (39% of input) in an ATP-dependent manner (Figure 3G). The amount of Rhp51 loaded under these conditions was much higher than with Swi5-Sfr1 alone or with Rad22 alone, indicating that the two mediators function synergistically to promote Rhp51 loading onto ssDNA. Interestingly, the nonhydrolyzable ATP analog, AMP-PNP, but not ATPγS, supported Rhp51 loading under these conditions. Small amounts of pulled-down Rhp51 were detected in the absence of nucleotide (Figure 3G, lane 2) and in the presence of ADP or ATPγS (Figure 3G, lanes 4 and 5). These low levels are likely due to Rhp51 associated with ssDNA through the Rad22–RPA interaction, as mentioned above.

### Rhp51 Displaces RPA from ssDNA

We next examined which protein factor is directly involved in displacing RPA from ssDNA (Figure 4). RPA was first incubated with css beads. ATP, Rhp51, and/or the two mediators were then added. The mixtures were pulled down, and proteins in the bound and unbound fractions were analyzed by SDS-PAGE. Neither Rad22 nor Swi5-Sfr1 could displace RPA from ssDNA (Figure 4A–4C). Only high amounts of Rhp51 could displace RPA at a detectable level, indicating that Rhp51 per se, but not the mediators, is an intrinsic factor that displaces RPA from ssDNA (Figure 4D). Swi5-Sfr1 stimulated Rhp51-dependent RPA displacement (Figure 4F), whereas Rad22 alone stimulated displacement only modestly (Figure 4E). See also the graphs of the amounts of displaced RPA (Figure 4H) and bound Rhp51 (Figure 4I). At low protein concentrations, Rad22 appeared to assist the loading of Rhp51 onto ssDNA at a considerably high level, as judged by the amount of Rhp51 in the bound fractions (Figure 4I). However, since the RPA levels in the unbound fractions increased only slightly when Rad22 was also included (Figure 4H), the observed increase of Rhp51 in Figure 4I may be due to Rhp51 bound to RPA-coated css beads via Rad22. It has been reported that Rad22 interacts with both Rhp51 and RPA [31,32], and the ternary complex can also be detected in vitro by coimmunoprecipitation (unpublished data). However, RPA displacement mediated by Rhp51 with Swi5-Sfr1 was further stimulated by coincubation with Rad22 (Figure 4G). This synergistic effect was robust; three independent experiments yielded the same results. Therefore, these results again indicate that the two mediators function coordinately to assist in the displacement of RPA. In the absence of ATP, RPA displacement did not increase from basal levels, even in the presence of all the protein components in a 4-fold excess relative to their concentrations in the standard reaction (unpublished data), indicating that RPA displacement requires ATP. This result is consistent with the hypothesis that Rhp51 per se is a displacing factor, since neither Rad22 (unpublished data) nor Swi5-Sfr1 is an ATP-binding protein [22].

**Figure 4:**
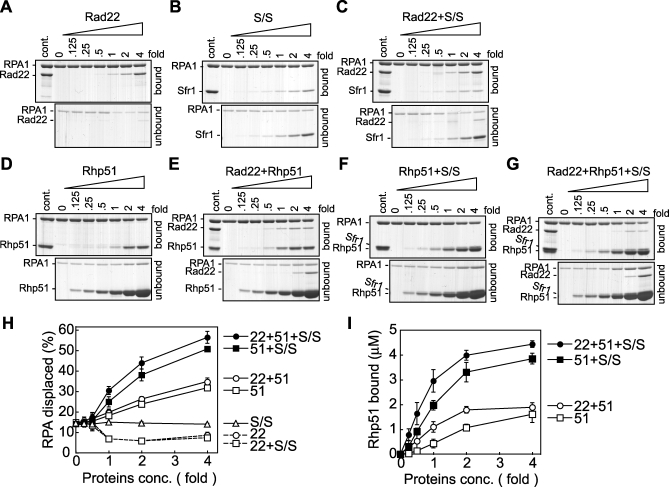
Rhp51 Per Se Is a Factor That Displaces RPA from ssDNA, and the Two Mediators Synergistically Facilitate This Reaction RPA-saturated ssDNA beads were prepared by incubating immobilized ssDNA with RPA (1 μM) and then removing excess RPA by washing the beads with a buffer. Varying concentrations of each protein (Rad22, Rhp51, and Swi5-Sfr1) or protein mixtures were incubated with RPA-saturated ssDNA in the presence of ATP (2 mM) at 37 °C for 30 min. The css beads were pulled down, and the bound and unbound fractions were analyzed by SDS-PAGE. The fold differences in protein concentrations, indicated above each gel, were based on the concentrations used in the standard strand exchange reaction in this study (i.e., “1-fold” corresponds to 5 μM Rhp51, 0.5 μM Swi5-Sfr1, and 0.5 μM Rad22). (A) Rad22 alone. (B) Swi5-Sfr1 (S/S) alone. (C) Rad22 plus Swi5-Sfr1. (D) Rhp51 alone. (E) Rhp51 plus Rad22. (F) Rhp51 plus Swi5-Sfr1. (G) Rhp51 plus Rad22 plus Swi5-Sfr1. (H) The graph shows the relative amounts of RPA (%) in the unbound fractions. Input RPA (bound + unbound) in the “0-fold sample” was normalized to 100%. (I) The graph shows the amounts of Rhp51 (in micromoles) in the bound fractions. The amount of Rhp51 in the bound fractions was estimated from lanes loaded with 2 μM Rhp51 as a standard. The values and error bars in the graphs are average percentages and s.d., respectively, obtained from three independent experiments. 22, Rad22; 51, Rhp51.

Time-course experiments for RPA displacement from and Rhp51 loading onto ssDNA were also performed (Figure 5). Rhp51 alone was not loaded onto ssDNA and did not promote RPA displacement (Figure 5A). A small amount of Rhp51 in the presence of Rad22 was loaded onto ssDNA, but this increase and the displacement of RPA ceased within 30 min (Figure 5B). Swi5-Sfr1 promoted Rhp51 loading and RPA displacement (Figure 5C), and coincubation of Rad22 and Swi5-Sfr1 strongly enhanced both processes (Figure 5D). A quantitative presentation of these assays is shown in Figure 5E and 5F. Taking these results together, we conclude that the two mediators work concertedly, but not exclusively, to promote Rhp51 loading onto and RPA displacement from ssDNA to form the active presynaptic filament required for effective DNA strand exchange.

**Figure 5:**
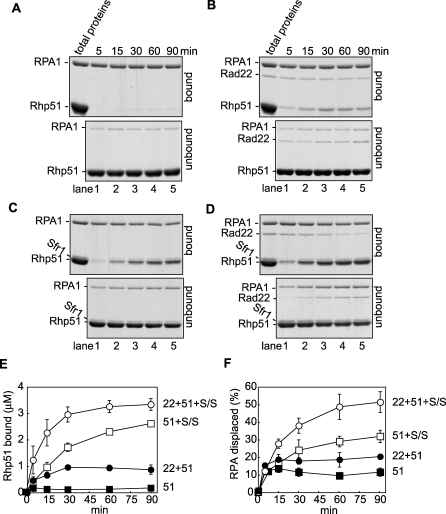
Time Course of Rhp51 Loading and RPA Displacement RPA-saturated css beads were prepared by incubating immobilized ssDNA with RPA and then removing excess RPA by washing the beads with a buffer. Protein mixtures containing Rhp51 (5 μM) were incubated with RPA-saturated ssDNA (approximately 10 μM nucleotide) in a final volume of 80 μl at time 0. Aliquots (12 μl) were taken at various time points, and the css beads were immediately pulled down. The bound and unbound fractions were analyzed by SDS-PAGE. The following proteins were incubated with RPA-saturated css beads: (A) Rhp51 (5 μM) alone; (B) Rhp51 (5 μM) and Rad22 (0.5 μM); (C) Rhp51 (5 μM) and Swi5-Sfr1 (0.5 μM); and (D) Rhp51 (5 μM), Rad22 (0.5 μM), and Swi5-Sfr1(0.5 μM). The graphs show the amount of Rhp51 (μM) in the bound fractions (E) and the relative amounts of RPA (%) in the unbound fractions (F). The values and error bars in the graphs are average percentages and s.d., respectively, obtained from three independent experiments. 22, Rad22; 51, Rhp51; S/S, Swi5-Sfr1.

### Rad22 Does Not Affect the ATPase Activity of Rhp51

The results described above suggest that the ATPase activity of Rhp51 plays an important role in both RPA displacement and Rhp51 filament formation. Therefore, we examined the ATPase activity of Rhp51 under various conditions (Figure 6). It has been reported that Rhp51 has low activity, but considerably higher than that of other Rad51 proteins in the absence of ssDNA and that this basal-level ATPase activity is very slightly enhanced in the presence of ssDNA [22,33]. We show here that neither of the two mediators has an effect on the intrinsic (DNA-free) or dsDNA-dependent ATPase activities of Rhp51 (Figure 6A and 6C). However, the Swi5-Sfr1 complex stimulated the ssDNA-dependent ATPase activity of Rhp51 about 3-fold (Figure 6B), consistent with a previous report [22], but Rad22 had no effect in this respect, regardless of the presence of the Swi5-Sfr1 complex (Figure 6B).

**Figure 6:**
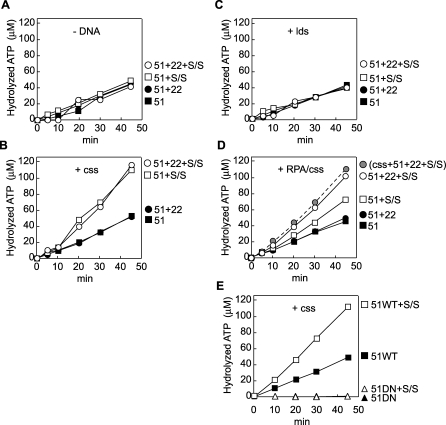
Effect of Mediators on the Rhp51 ATPase Activity Swi5-Sfr1, but not Rad22, specifically activates the ssDNA-dependent ATPase activity of Rhp51. Proteins, ssDNA, or dsDNA, as indicated, were mixed in different combinations at the concentrations used in the standard strand exchange reaction. (A–D) Time courses of ATP hydrolysis are shown in the absence of (A) DNA or the presence of (B) ssDNA, (C) dsDNA, or (D) RPA-coated ssDNA. Note that the gray circles in (D) indicate a reaction containing all protein components in the presence of RPA-free css, used as a control, essentially the same as the open circles in (B). (E) The Walker B box mutant protein Rhp51D244N is defective in ATP hydrolysis, and Swi5-Sfr1 does not stimulate its ATPase activity. 22, Rad22; 51, Rhp51; S/S, Swi5-Sfr1.

### Swi5-Sfr1 Renders Rhp51 Resistant to RPA Attack in an ATP-Dependent Manner

We hypothesized that stimulation of the ssDNA-dependent ATPase activity of Rhp51 by Swi5-Sfr1 alters the Rhp51 filament. An RPA attack experiment supported this hypothesis (Figure 7A–7E). Rhp51 filaments were allowed to form on css beads under various conditions, and RPA was then added. Proteins that remained bound to ssDNA were analyzed by SDS-PAGE. In the presence of ATP, but absence of RPA, Rhp51 was pulled down efficiently (Figure 7A, lanes 1–3, and Figure 3C). Swi5-Sfr1 had no effect on the ssDNA binding capacity of Rhp51, as previously reported [22]. However, when RPA was added to the reaction mixture in the absence of Swi5-Sfr1, it became detectable in the ssDNA fraction, and almost all of the Rhp51 bound to ssDNA disappeared (Figure 7A, lane 4). In contrast, when RPA was added to the reaction mixture in the presence of Swi5-Sfr1, the Rhp51 filament became resistant to RPA attack in a Swi5-Sfr1 concentration-dependent manner (Figure 7A, lanes 5 and 6). The formation of the resistant Rhp51 filament was ATP dependent, and neither ADP nor ATPγS could replace ATP in this reaction (Figure 7B–7D). Interestingly, AMP-PNP made the Rhp51 filament resistant to RPA attack, even in the absence of Swi5-Sfr1 (Figure 7E), consistent with a report that human Rad51 forms more stable filaments with AMP-PNP [34,35].

**Figure 7:**
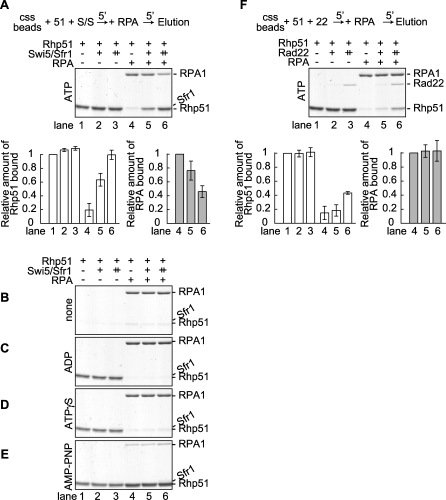
Swi5-Sfr1 Activates the Rhp51 Filament in an ATP-Dependent Manner The css beads were mixed with 5 μM Rhp51 in the presence or absence of mediators at 37 °C for 5 min, followed by the addition of RPA. After incubation at 37 °C for 5 min, proteins bound to the beads were pulled down and analyzed by SDS-PAGE. (A) Upper panel, SDS-PAGE gel of the pulled down samples in the presence of ATP. Lower panel, graphs showing the amounts of Rhp51 (51) and RPA bound to ssDNA, determined from their band intensities in the gel. S/S, Swi5-Sfr1. (B) Rhp51 cannot bind to ssDNA in the absence of adenine nucleotide. (C) In the presence of ADP, Rhp51 binds to ssDNA, but RPA displaces Rhp51 from ssDNA, even in the presence of Swi5-Sfr1. (D) In the presence of ATPγS, the results are similar to those obtained with ADP. (E) In the presence of AMP-PNP, Rhp51 binds to ssDNA as in the presence of other nucleotides (lanes 1–3). Even in the absence of Swi5-Sfr1, Rhp51 is resistant to the RPA challenge (lane 4). (F) An experiment similar to (A) was performed with Rad22 (22) instead of Swi5-Sfr1. The values and error bars in the graphs in (A) and (F) are average percentages and s.d., respectively, obtained from three independent experiments. (A–F), the plus sign (+) indicates the presence of Rhp51 (51; 5 μM), Swi5-Sfr1 (0.1 μM), RPA (1 μM), and Rad22 (0.1 μM), and each double plus sign (++) indicates both Swi5-Sfr1 (0.25 μM) and Rad22 (0.25 μM).

Rad22 had little detectable effect on the formation of resistant Rhp51 filaments (Figure 7F). However, large amounts of Rad22 modestly facilitated Rhp51 pull down (Figure 7F, lane 6). Unlike what was seen for coincubation with Swi5-Sfr1 (Figure 7A), however, the amount of RPA bound to ssDNA was constant (Figure 7F, lanes 4–6, and the histogram below). In addition, the recovery of Rhp51 was adenine nucleotide-independent (unpublished data). Therefore, Rhp51 recovered with assistance of Rad22 is not the same as the resistant Rhp51 induced by Swi5-Sfr1. Since Rad22 binds strongly to both Rhp51 and RPA (unpublished data, and [31,32]), and the ternary complex is formed in solution in the absence of ATP (unpublished data), these results indicate that an RPA-Rad22-Rhp51 complex bound to ssDNA via RPA is pulled down.

Taken together, these results suggest that Swi5-Sfr1 induces activation of the Rhp51 filament to promote strand exchange in an ATP-dependent manner. Rad22 may not be directly involved in filament activation, but rather, it may recruit Rhp51 to RPA-coated ssDNA.

### The Two Mediators Synergistically Stimulate the Rhp51 ATPase Activity in the Presence of RPA-Coated ssDNA

We measured the ATPase activity of Rhp51 using RPA-coated ssDNA as a cofactor, which more closely approximates physiological conditions. As shown in Figure 6D, Swi5-Sfr1 stimulated the ATPase activity of Rhp51, but this stimulation was less than that observed when RPA-free ssDNA was used as a cofactor (compare Figure 6B and 6D). Interestingly, the level of stimulation of the Rhp51 ATPase activity by Swi5-Sfr1 reached the level of that observed with RPA-free ssDNA when Rad22 was added to the reaction (Figure 6D). This synergistic stimulation of Rhp51 ATPase activity is consistent with the coordinated action of these two mediators in DNA strand exchange. In a control experiment, we constructed and purified an Rhp51 Walker B box mutant protein (D to N alteration; Rhp51D244N). The behavior of Rhp51D244N during purification by chromatography was the same as that of the wild-type protein (unpublished data). The mutant did not produce any detectable levels of phosphate generated by ATP hydrolysis. Coincubation of Swi5-Sfr1 with Rhp51D244N did not increase the level of hydrolyzed phosphate (Figure 6E). This result indicates that the ATPase activity stimulated by Swi5-Sfr1 is indeed that of the wild-type Rhp51 protein.

## Discussion

This study demonstrates an in vitro reconstitution of the central step in eukaryotic HR. Our system consists entirely of purified components, including recombinase, RPA, and the Rad22 and Swi5-Sfr1 mediators, and it reflects the genetic requirements for these components in vivo. Using this system, we present for the first time robust biochemical evidence that the two mediators function in a concerted manner to form the active Rhp51 filament. Dissection of the reaction uncovered several of the molecular details of strand exchange. First, Rad22 overcomes the inhibitory effect of RPA, but not of SSB, in strand exchange mediated by Rhp51-Swi5-Sfr1 (Figure 1B). In addition, Rad22 negates the need for a strict order of addition of protein components, indicating that it coordinates strand exchange (Figure 2). However, even in the presence of Rad22, Swi5-Sfr1 is essential for Rhp51-mediated strand exchange, highlighting the different fundamental properties of the two mediators (Figure 1C). Although the molecular functions of the two mediators are distinct (see below), they function synergistically to promote Rhp51 loading (Figure 3). Importantly, Rhp51, but not Rad22 or the Swi5-Sfr1 mediator, displaces RPA from ssDNA (Figures 4 and 5). We previously showed that Swi5-Sfr1 stimulates the ssDNA-dependent ATPase activity of Rhp51 [22]. Here, we demonstrated that Rad22 does not affect the ATPase activity of Rhp51 (Figure 6). Most important, Swi5-Sfr1 renders Rhp51 resistant to RPA attack in an ATP-dependent manner (Figure 7A), suggesting that Swi5-Sfr1 stabilizes the presynaptic filaments of the recombinase. Since this stabilization is ATP-dependent, Swi5-Sfr1 stimulates the ssDNA-dependent ATPase activity of Rhp51, and the mediators stimulate strand exchange mediated by Rhp51. Thus, these mutual relationships strongly suggest that the induced stabilization of the Rhp51 filament reflects a structural/functional alteration upon activation. In contrast, Rad22 is not involved in Rhp51 activation (Figure 7F). Based on these results, we propose a model for the early step of the strand exchange reaction involving Rhp51 and the two mediators. Rad22 recruits Rhp51 to RPA-coated ssDNA. Rad22 and Swi5-Sfr1 collaborate in displacing RPA and loading Rhp51 onto ssDNA, but the displacing factor itself is Rhp51, and displacement requires the ATP binding activity of Rhp51. Swi5-Sfr1 activates Rhp51 recruited to RPA by Rad22 to form and stabilize/activate the presynaptic filament in an ATP-dependent manner, promoting processive strand exchange.

Localization of budding yeast Rad51 to DSB sites requires Rad52 [5,6,26,36,37]. Although experimental data on whether Rhp51 focus formation is dependent on Rad22 functions are not yet available, at least to our knowledge, this is widely believed to be true as well for fission yeast, based on similarities between the two systems. However, the contribution of Rad22 to our in vitro strand exchange reaction was relatively small. In contrast, the influence of the other mediator, Swi5-Sfr1, is much stronger than that of Rad22. Our results indicate that substoichiometric amounts of Rad22 are optimal to overcome the RPA inhibitory effect on strand exchange (1 to 5∼10 ratio of Rad22 to Rhp51; see Figure 2D). This is similar to what has been seen for budding yeast Rad52 in strand exchange [38]. These results suggest that Rad22 may only transiently interact with the Rhp51 nucleoprotein filament. Such transient interactions likely mediate assembly of the Rhp51 filament. However, Rad22 (and presumably Rad52) may have (an)other uncovered function(s) involved in the overall in vivo strand exchange reaction. Based on chromatin immunoprecipitation analyses, Wolner and coworkers suggested that Rad51 binds first, followed by Rad52 and Rad55, to a single DSB site in budding yeast [26]. This result apparently contradicts the genetic dependency of Rad51 focus formation on Rad52. Wolner et al. proposed that only the stable binding of Rad51 is detectable by chromatin immunoprecipitation, reflecting the assembly of a nucleoprotein filament catalytically competent for strand invasion. Indeed, Arai et al. have demonstrated that a stoichiometric complex of Rad52 with Rad51 is required for the efficient formation of D-loops via strand invasion [39]. The strand exchange system we used in this study does not include a strand invasion step, accounting for the small amounts of optimal concentration and a lower dependency on Rad22. An apparent strong dependency on Swi5-Sfr1 may be valid only for a three-strand exchange reaction using a long DNA substrate, an assay that mimics the strand transfer reaction but which does not include strand invasion. Further study will be needed to reveal functional differences between the two mediators in D-loop formation.

We propose here a two-phase activation mechanism of Rhp51 filament formation. Rhp51 is the only component with ATPase activity among the proteins used in our assay, and this activity enables the protein to bind to ssDNA in an adenine nucleotide-dependent manner. However, Rhp51 alone cannot efficiently promote strand exchange; instead, it requires Swi5-Sfr1 [22]. Thus, the first phase of activation is one in which Rhp51 is activated to bind ssDNA. Swi5-Sfr1 then further activates ATP-bound Rhp51 to make it catalytically competent for strand exchange. In the second phase of activation, the Swi5-Sfr1 mediator also renders the Rhp51 filament resistant to attack by RPA (Figure 7A).

The ATPase activities of Rad51 proteins from budding yeast and human cells are dependent on the presence of ssDNA [40–42]. In contrast, the Rhp51 ATPase is considerably efficient in the absence of ssDNA, and the presence of ssDNA does not enhance its activity. Binding of human Rad51 to DNA can occur in the absence of ATP, but budding yeast Rad51 requires ATP for DNA binding [43]. Rhp51 also requires adenine nucleotides for ssDNA binding (Figure 3). These observations indicate that Rad51 properties relevant to ATPase and DNA binding are not universal, and variations may be related to the apparent differences in activation mechanisms for strand exchange. Budding yeast and human Rad51 proteins can carry out a robust strand exchange reaction in the absence of a Swi5-Sfr1-type mediator in vitro. Human Rad51 is already activated for the first step because it can bind ssDNA without ATP. Once ATP is included in the reaction, human Rad51 may be further activated to the catalytically competent state. Budding yeast Rad51 requires ATP to bind DNA, implying that the first stage is similar to that of Rhp51. Once Rad51 binds to ssDNA, its ATPase activity is stimulated, indicating that the second phase of activation readily occurs without a Swi5-Sfr1–type mediator. In other words, the basal level of activation of the first phase is higher in human Rad51, and the second phase is higher in budding yeast Rad51. This idea can reconcile the apparent differences among the three recombinases.

Interestingly, under biochemical conditions that slow ATPase activity, such as the presence of Ca^2+^, strand exchange mediated by human Rad51 is enhanced [34,44,45]. However, activation by Ca^2+^ holds only for human Rad51, not for budding yeast Rad51 [44] or fission yeast Rhp51 (unpublished data). The presence of Ca^2+^ attenuates the disassembly of Rad51 from ssDNA, resulting in stable filaments on ssDNA [45]. In contrast, Swi5-Sfr1 stimulates the Rhp51 ATPase [22] and stabilizes the filament in an ATP-dependent manner (Figure 7). We cannot explain why these two opposing effects lead to a stimulation of strand exchange, although the differences in the basal-level status of yeast and human Rad51 proteins may be a key to this issue.

AMP-PNP, but not ATPγS, induces a similar stabilization of Rhp51 that is independent of Swi5-Sfr1. Although Rhp51 binding to AMP-PNP is thought to mimic the transient ATP-bound form during ATP hydrolysis, the AMP-PNP–bound and ATP-bound Rhp51 forms are qualitatively different. The AMP-PNP–bound form of Rhp51 must be locked in a unique activated state with Swi5-Sfr1, which presumably represents the second phase of activation, since the ATP-bound Rhp51 filament itself is not sufficient for strand exchange. Importantly, AMP-PNP cannot support processive strand exchange. Both the Swi5-Sfr1–activated ATP-bound form and the AMP-PNP–bound form are competent states that promote homologous pairing and the transient formation of a three-strand intermediate. However, hydrolysis of ATP is required for the subsequent steps of consecutive strand transfer from duplex DNA to ssDNA that result in the formation of a long heteroduplex. Swi5-Sfr1 promotes both of the states that permit homologous pairing and consecutive strand transfer by stimulating the ssDNA-dependent ATPase activity of Rhp51. The enhancement of the Rhp51 ATPase activity by Swi5-Sfr1 is not due to an increased ADP–ATP exchange rate, but rather to an enhanced turnover rate [22]. A rapid turnover between the first and the second stages might promote efficient strand exchange. The precise mechanism by which Swi5-Sfr1 induces activation of the Rhp51 filament remains to be clarified in future studies.

## Materials and Methods

### Proteins.

S. pombe Rhp51 was purified as previously described [22]. Alternatively, Rhp51 was expressed in the Escherichia coli strain BL21-CodonPlus(DE3)-RIPL carrying an Rhp51 expression plasmid derivative of pET11b (Novagen). Cells were incubated at 37 °C in LB media containing ampicillin. When cell density reached an optical density at 600 nm (OD_600_) of approximately 0.5, isopropyl-ß-D-thiogalactopyranoside (IPTG) was added to a final concentration of 0.5 mM, and the culture was further incubated for 18 h at 18 °C. The cells were collected by centrifugation and resuspended in R buffer (20 mM Tris-HCl [pH 8.0], 1 mM EDTA, 1 mM dithiothreitol [DTT], 10% glycerol) containing 300 mM NaCl. The cells were disrupted by sonication, and the lysate was clarified by ultracentrifugation. Proteins in the lysate were precipitated by ammonium sulfate fractionation at 40% saturation and centrifuged 35,000 × *g* for 30 min. The pellet was resuspended in P buffer (20 mM potassium phosphate [pH 7.5], 0.5 mM EDTA, 10% glycerol, and 0.5 mM DTT) containing 300 mM KCl and diluted by P buffer to a final concentration of KCl 50 mM before being subjected to SP Sepharose (GE Healthcare) chromatography. The pass-through fraction was collected and directly subjected to Q Sepharose (GE Healthcare) chromatography. The proteins were eluted with a linear gradient of 50 mM to 800 mM KCl in P buffer. Rhp51 was eluted at approximately 500 mM KCl. The peak fractions were diluted 5-fold with P buffer and loaded onto a HiTrap Heparin column (GE Healthcare). Rhp51 was eluted at approximately 400 mM KCl in a linear gradient of 100 mM to 700 mM KCl in P buffer. The peak fractions were diluted 4-fold with P buffer and loaded onto a Resource Q column (GE Healthcare). Rhp51 was then eluted at approximately 500 mM of KCl in a linear gradient of 100 mM to 600 mM KCl in P buffer. The Rhp51 preparation obtained by this procedure is indistinguishable from that obtained by a previously described method [22].

An *rhp51* derivative with a mutated Walker B box (*rhp51* D244N) was constructed by site-directed mutagenesis using a QuickChange kit (Stratagene). The sequences of the primer set are 5′-CATTGTTAGTTGTCaATAGTTGTACTGCC-3′ and 5′-GGCAGTACAACTATtGACAACTAACAATG-3′, where the lowercase letters are mutations that convert D to N. The mutant gene was subcloned into pET11b and expressed in the same manner as wild-type *rhp51*, and *rhp51* D244N was purified as described above. The chromatographic elution patterns of Rhp51D244N were the same as for wild-type Rhp51.

S. pombe Rad22 was also expressed in E. coli BL21-CodonPlus(DE3)-RIPL carrying a Rad22 expression plasmid derivative of pET11b (Novagen). Rad22 expression was induced by 0.2 mM IPTG at 30 °C for 3 h. The induced cell lysate was processed as described above, and the clarified lysate in R buffer containing 500 mM NaCl was precipitated by ammonium sulfate (30% saturation). The pellet was resuspended in R buffer containing 200 mM NaCl and directly loaded onto a Q Sepharose column, and Rad22 was eluted in one step with 600 mM NaCl in R buffer. Rad22 fractions were collected, diluted 3-fold with R buffer and loaded onto a HiTrap Heparin column. Rad22 was eluted at 500 mM NaCl with a linear gradient of 200 mM to 600 mM NaCl in R buffer. Peak fractions were collected and diluted 5-fold with R buffer and loaded onto a HiTrap SP column (GE Healthcare). A linear gradient of 100 mM to 600 mM NaCl in R buffer allowed elution of Rad22 at approximately 300 mM NaCl. Rad22 fractions were applied to a Superdex 16/60 200 pg column (GE Healthcare) and developed in R buffer containing 1 M NaCl. Rad22 was eluted in the void fractions and dialyzed against R buffer containing 100 mM NaCl. The dialyzed sample was applied to a Resource Q column. A linear gradient of 100 mM to 700 mM NaCl in R buffer allowed elution of Rad22 at approximately 250 mM NaCl.

Protein concentrations were determined by measuring absorbance at 280 nm. The following extinction coefficients (ɛ280) were used: 1.86 × 10^4^ M^−1^ cm^−1^ for Rhp51 and Rhp51D244N, 2.93 × 10^4^ M^−1^ cm^−1^ for Rad22, 1.44 × 10^4^ M^−1^ cm^−1^ for the Swi5-Sfr1 complex, and 9.89 × 10^4^ M^−1^ cm^−1^ for RPA.

The purification of S. pombe RPA and the Swi5-Sfr1 complex was previously described [22]. E. coli SSB was purchased from Sigma-Aldrich.

### Three-strand DNA exchange reaction.

Procedures for the standard reaction protocols were essentially the same as previously described [22], with the exception of Rad22 addition. Briefly, the reactions (10 μl) contained the following components: 10 μM øX174 ssDNA (css), 10 μM ApaLI-linearized øX174 dsDNA (lds) (New England Biolabs), 5 μM Rhp51, 0.5 μM Rad22, 0.5 μM Swi5-Sfr1, 1 μM RPA, 2 mM ATP, and an ATP regeneration system (8 mM creatine phosphate and 8 U/ml creatine kinase) in buffer F (25 mM Tris-OAc [pH 7.5], 1 mM DTT, 5% glycerol, 3 mM Mg(OAc)_2_, 100 mM KCl). When replacing RPA, SSB was used at 2 μM. Reactions were incubated for 120 min at 37 °C and terminated by adding 1.2 μl of a stop solution containing 8% SDS and 0.6 μl 20 mg/ml Proteinase K, with a final incubation for 30 min at 37 °C. The products were analyzed by 1% agarose gel electrophoresis as previously described [22].

### Pull-down assay for DNA binding.

Immobilized øX174 ssDNA beads (css beads) were prepared by annealing a 5′-biotinylated 100-mer oligonucleotide to øX174 ssDNA and capturing the fragments with Dynabeads M-280 Streptavidin (Invitrogen), as previously described [22,46]. To determine the amount of ssDNA immobilized on the beads, an aliquot of the css-beads suspension was denatured by 0.1 M NaOH, and the concentration of the released ssDNA was determined by measuring at A_260_. About 80% of css was immobilized on the beads. In a standard assay, a bead suspension (2 μl) containing 33 ng of css was mixed with the indicated amounts of each protein in the presence or absence of nucleotide in 10 μl of buffer F containing 0.01% (v/v) NP-40 for 30 min at 37 °C, with constant tapping. The beads were captured with a Magnet Stand Dynal MPC (Invitrogen), and the supernatants (the unbound fraction) and beads (bound fraction) were separated. The bead-bound proteins were eluted with 15 μl of SDS-PAGE loading buffer, and 12 μl of the eluates was analyzed by SDS-PAGE. A 5-fold concentration of SDS loading buffer (3 μl) was added to the supernatants, and 12 μl of each sample was analyzed by SDS-PAGE. The gels were stained with BioSafe CBB G-250 (Bio-Rad), gel images were captured by LAS-4000 (Fuji Photo Film), and protein band densities were quantified with Multi Gauge (Fuji Photo Film) to determine the amounts of bound and unbound proteins.

### ATPase assay.

The procedures were conducted essentially as previously described [22]. Reaction mixtures (13.5 μl) contained 5 μM Rhp51 in buffer F. In some assays, 1 μM RPA, 0.5 μM Swi5-Sfr1, 0.5 μM Rad22, 10 μM øX174 ssDNA, or 10μM ApaL1-linearized øX174 dsDNA were added, as indicated. The reactions were started by adding 1.5 μl of a mixture of [γ-^32^P]ATP and cold ATP (final concentration, 2 mM) at 37 °C. Aliquots (2 μl) were taken at various time points and immediately mixed with 4 μl of stop solution (0.5 M EDTA). Samples (1 μl) were subjected to thin layer chromatography, as previously described [22]. The amounts of ^32^Pi and [γ-^32^P]ATP in each spot were determined using a phosphoimager (Fuji BAS2500).

## Abbreviations

css: circular single-stranded DNA
DSB: double-stranded break
dsDNA: double-stranded DNA
HR: homologous recombination
JM: joint molecule
lds: linear double-stranded DNA, NC, nicked circular DNA
RPA: replication protein A
SSB: single-stranded DNA binding protein
ssDNA: single-stranded DNA

## References

[pbio-0060088-b001] Pierce AJ, Stark JM, Araujo FD, Moynahan ME, Berwick M (2001). Double-strand breaks and tumorigenesis. Trends Cell Biol.

[pbio-0060088-b002] Symington LS (2002). Role of RAD52 epistasis group genes in homologous recombination and double-strand break repair. Microbiol Mol Biol Rev.

[pbio-0060088-b003] Sung P, Krejci L, Van Komen S, Sehorn MG (2003). Rad51 recombinase and recombination mediators. J Biol Chem.

[pbio-0060088-b004] Krogh BO, Symington LS (2004). Recombination proteins in yeast. Annu Rev Genet.

[pbio-0060088-b005] Lisby M, Barlow JH, Burgess RC, Rothstein R (2004). Choreography of the DNA damage response: spatiotemporal relationships among checkpoint and repair proteins. Cell.

[pbio-0060088-b006] Miyazaki T, Bressan DA, Shinohara M, Haber JE, Shinohara A (2004). In vivo assembly and disassembly of Rad51 and Rad52 complexes during double-strand break repair. EMBO J.

[pbio-0060088-b007] Sung P (1997). Yeast Rad55 and Rad57 proteins form a heterodimer that functions with replication protein A to promote DNA strand exchange by Rad51 recombinase. Genes Dev.

[pbio-0060088-b008] Fortin GS, Symington LS (2002). Mutations in yeast Rad51 that partially bypass the requirement for Rad55 and Rad57 in DNA repair by increasing the stability of Rad51-DNA complexes. EM J.

[pbio-0060088-b009] Gasior SL, Olivares H, Ear U, Hari DM, Weichselbaum R (2001). Assembly of RecA-like recombinases: distinct roles for mediator proteins in mitosis and meiosis. Proc Natl Acad Sci U S A.

[pbio-0060088-b010] Sigurdsson S, Van Komen S, Bussen W, Schild D, Albala JS (2001). Mediator function of the human Rad51B-Rad51C complex in Rad51/RPA-catalyzed DNA strand exchange. Genes Dev.

[pbio-0060088-b011] Rodrigue A, Lafrance M, Gauthier MC, McDonald D, Hendzel M (2006). Interplay between human DNA repair proteins at a unique double-strand break in vivo. EMBO J.

[pbio-0060088-b012] Yang H, Li Q, Fan J, Holloman WK, Pavletich NP (2005). The BRCA2 homologue Brh2 nucleates RAD51 filament formation at a dsDNA-ssDNA junction. Nature.

[pbio-0060088-b013] San Filippo J, Chi P, Sehorn MG, Etchin J, Krejci L (2006). Recombination mediator and Rad51 targeting activities of a human BRCA2 polypeptide. J Biol Chem.

[pbio-0060088-b014] Davies OR, Pellegrini L (2007). Interaction with the BRCA2 C terminus protects RAD51-DNA filaments from disassembly by BRC repeats. Nat Struct Mol Biol.

[pbio-0060088-b015] Esashi F, Galkin VE, Yu X, Egelman EH, West SC (2007). Stabilization of RAD51 nucleoprotein filaments by the C-terminal region of BRCA2. Nat Struct Mol Biol.

[pbio-0060088-b016] Haruta N, Akamatsu Y, Tsutsui Y, Kurokawa Y, Murayama Y (2008). Fission yeast Swi5 protein, a novel DNA recombination mediator. DNA Repair (Amst).

[pbio-0060088-b017] Akamatsu Y, Dziadkowiec D, Ikeguchi M, Shinagawa H, Iwasaki H (2003). Two different Swi5-containing protein complexes are involved in mating-type switching and recombination repair in fission yeast. Proc Natl Acad Sci U S A.

[pbio-0060088-b018] Akamatsu Y, Tsutsui Y, Morishita T, Siddique MS, Kurokawa Y (2007). Fission yeast Swi5/Sfr1 and Rhp55/Rhp57 differentially regulate Rhp51-dependent recombination outcomes. EMBO J.

[pbio-0060088-b019] Ellermeier C, Schmidt H, Smith GR (2004). Swi5 acts in meiotic DNA joint molecule formation in Schizosaccharomyces pombe. Genetics.

[pbio-0060088-b020] Hayase A, Takagi M, Miyazaki T, Oshiumi H, Shinohara M (2004). A protein complex containing Mei5 and Sae3 promotes the assembly of the meiosis-specific RecA homolog Dmc1. Cell.

[pbio-0060088-b021] Tsubouchi H, Roeder GS (2004). The budding yeast Mei5 and Sae3 proteins act together with dmc1 during meiotic recombination. Genetics.

[pbio-0060088-b022] Haruta N, Kurokawa Y, Murayama Y, Akamatsu Y, Unzai S (2006). The Swi5-Sfr1 complex stimulates Rhp51/Rad51 - and Dmc1-mediated DNA strand exchange in vitro. Nat Struct Mol Biol.

[pbio-0060088-b023] Sung P (1997). Function of yeast Rad52 protein as a mediator between replication protein A and the Rad51 recombinase. J Biol Chem.

[pbio-0060088-b024] Shinohara A, Ogawa T (1998). Stimulation by Rad52 of yeast Rad51-mediated recombination. Nature.

[pbio-0060088-b025] New JH, Sugiyama T, Zaitseva E, Kowalczykowski SC (1998). Rad52 protein stimulates DNA strand exchange by Rad51 and replication protein A. Nature.

[pbio-0060088-b026] Wolner B, van Komen S, Sung P, Peterson CL (2003). Recruitment of the recombinational repair machinery to a DNA double-strand break in yeast. Mol Cell.

[pbio-0060088-b027] Sugiyama T, Kowalczykowski SC (2002). Rad52 protein associates with replication protein A (RPA)-single-stranded DNA to accelerate Rad51-mediated displacement of RPA and presynaptic complex formation. J Biol Chem.

[pbio-0060088-b028] Shen Z, Cloud KG, Chen DJ, Park MS (1996). Specific interactions between the human RAD51 and RAD52 proteins. J Biol Chem.

[pbio-0060088-b029] Park MS, Ludwig DL, Stigger E, Lee SH (1996). Physical interaction between human RAD52 and RPA is required for homologous recombination in mammalian cells. J Biol Chem.

[pbio-0060088-b030] Jackson D, Dhar K, Wahl JK, Wold MS, Borgstahl GE (2002). Analysis of the human replication protein A:Rad52 complex: evidence for crosstalk between RPA32, RPA70, Rad52 and DNA. J Mol Biol.

[pbio-0060088-b031] Tsutsui Y, Khasanov FK, Shinagawa H, Iwasaki H, Bashkirov VI (2001). Multiple interactions among the components of the recombinational DNA repair system in Schizosaccharomyces pombe. Genetics.

[pbio-0060088-b032] Kim WJ, Park EJ, Lee H, Seong RH, Park SD (2002). Physical interaction between recombinational proteins Rhp51 and Rad22 in Schizosaccharomyces pombe. J Biol Chem.

[pbio-0060088-b033] Sauvageau S, Stasiak AZ, Banville I, Ploquin M, Stasiak A (2005). Fission yeast Rad51 and Dmc1, two efficient DNA recombinases forming helical nucleoprotein filaments. Mol Cell Biol.

[pbio-0060088-b034] Chi P, Van Komen S, Sehorn MG, Sigurdsson S, Sung P (2006). Roles of ATP binding and ATP hydrolysis in human Rad51 recombinase function. DNA Repair (Amst).

[pbio-0060088-b035] Raschle M, Van Komen S, Chi P, Ellenberger T, Sung P (2004). Multiple interactions with the Rad51 recombinase govern the homologous recombination function of Rad54. J Biol Chem.

[pbio-0060088-b036] Gasior SL, Wong AK, Kora Y, Shinohara A, Bishop DK (1998). Rad52 associates with RPA and functions with rad55 and rad57 to assemble meiotic recombination complexes. Genes Dev.

[pbio-0060088-b037] Sugawara N, Wang X, Haber JE (2003). In vivo roles of Rad52, Rad54, and Rad55 proteins in Rad51-mediated recombination. Mol Cell.

[pbio-0060088-b038] Song B, Sung P (2000). Functional interactions among yeast Rad51 recombinase, Rad52 mediator, and replication protein A in DNA strand exchange. J Biol Chem.

[pbio-0060088-b039] Arai N, Ito D, Inoue T, Shibata T, Takahashi H (2005). Heteroduplex joint formation by a stoichiometric complex of Rad51 and Rad52 of Saccharomyces cerevisiae. J Biol Chem.

[pbio-0060088-b040] Sung P (1994). Catalysis of ATP-dependent homologous DNA pairing and strand exchange by yeast RAD51 protein. Science.

[pbio-0060088-b041] Benson FE, Stasiak A, West SC (1994). Purification and characterization of the human Rad51 protein, an analogue of E. coli RecA. EMBO J.

[pbio-0060088-b042] Gupta RC, Bazemore LR, Golub EI, Radding CM (1997). Activities of human recombination protein Rad51. Proc Natl Acad Sci U S A.

[pbio-0060088-b043] De Zutter JK, Knight KL (1999). The hRad51 and RecA proteins show significant differences in cooperative binding to single-stranded DNA. J Mol Biol.

[pbio-0060088-b044] Bugreev DV, Mazin AV (2004). Ca^2+^ activates human homologous recombination protein Rad51 by modulating its ATPase activity. Proc Natl Acad Sci U S A.

[pbio-0060088-b045] van der Heijden T, Seidel R, Modesti M, Kanaar R, Wyman C (2007). Real-time assembly and disassembly of human RAD51 filaments on individual DNA molecules. Nucleic Acids Res.

[pbio-0060088-b046] Sigurdsson S, Trujillo K, Song B, Stratton S, Sung P (2001). Basis for avid homologous DNA strand exchange by human Rad51 and RPA. J Biol Chem.

